# Contemporary aetiology of acute heart failure in a teaching hospital in Ghana

**DOI:** 10.1186/s12872-023-03103-3

**Published:** 2023-02-10

**Authors:** Francis Agyekum, Aba A. Folson, Bernard Yeboah-Asiamah Asare, Alfred Doku, John Kpodonu, Rafiq Okine, Forster Fokuoh, Joseph Atiah Akamah

**Affiliations:** 1 Department of Medicine, College of Health Sciences, Korlebu Teaching Hospital, University of Ghana, Accra, Ghana; 2 University of Health and Allied Sciences, Ho, Ghana; 3 Curtin School of Population Health, Curtin University, Kent Street, Perth, 6102 Australia; 4 Health Psychology, Institute of Applied Health Sciences, University of Aberdeen, Aberdeen, AB25 2ZD UK; 5 World Health Organization, Country Office for Ghana, Accra, Ghana; 6 Eastern Regional Hospital, P. O. Box 201, Koforidua, Ghana

**Keywords:** Aetiologies, Cardiovascular risk factors, Heart failure, Ghana

## Abstract

**Background:**

Heart failure (HF) is recognized as a global public health disease associated with high morbidity and mortality. It is suggested that the main underlying causes of HF in developing countries differ from those identified in well-resourced countries**.** This study therefore presents the cardiovascular risk factors and the underlying aetiology of HF among admitted patients in a teaching Hospital in Ghana.

**Method:**

The study prospectively recruited 140 consecutive patients admitted for heart failure at the Medical department of the Korle-Bu Teaching Hospital from March to October, 2014. The study evaluated the cardiovascular risk factors and the aetiologies of heart failure, and compared the risk factors and aetiologies with patient’s age and gender.

**Results:**

The mean age of the study participants was 51.3 ± 16.8 years. The commonest cardiovascular risk factors observed were hypertension (46.5%), history of previous HF (40.7%), excessive alcohol use (38.6%), and family history of heart disease (29.3%); predominantly hypertension (68.3%). The major underlying aetiology of HF were dilated cardiomyopathy (38.6%), hypertensive heart disease (21.4%), ischaemic heart disease (13.6%) and valvular heart disease (12.9%). These underlying aetiology of HF were more common in patients aged 40 years and above (*p* = 0.004) and those presenting with multiple risk factors (*p* = 0.001).

**Conclusion:**

The major underlying aetiology of heart failure in adults were dilated cardiomyopathy, hypertensive heart disease, ischaemic heart disease and valvular heart disease, which were significantly high among patients aged 40 years and above and those presenting multiple risk factors. Hypertension, excessive alcohol use, family history of heart disease and personal history of previous heart failure diagnosis are noted as the main cardiovascular risk factors among heart failure patients.

## Background

Heart failure (HF) has been singled out as an emerging epidemic [1–3]. In developing countries, the main underlying causes of HF are suggested to be different from those identified in well-resourced countries [4–7]. About 90% of HF in sub-Saharan Africa are attributed mainly to hypertensive heart disease, cardiomyopathy, rheumatic heart disease, congenital heart disease and pericardial diseases [8]. Cardiovascular disease is common in the general population and resulted in 17.3 million deaths worldwide in 2013 [9]. Several risk factors have been identified as contributing to the initial myocardial injury that eventually results in chronic heart failure and/or sudden cardiac death through mechanisms including endothelial dysfunction, cardiac remodeling, neurohormonal adaptations, diastolic and systolic dysfunction [10].

HF is a common terminal condition among many common chronic cardiovascular diseases in Ghana and accounted for about 8% of all admissions [11] and also responsible for about 10% of mortalities at the department of medicine in Korle-Bu Teaching Hospital in the early 1970s [12]. However, only a few hospital-based studies have characterized the aetiological factors of HF [13–15], and recent data on acute HF are limited in Ghana and in developing countries as a whole. This gap in research can potentially mitigate against efforts at prevention, control and management and there is thus an urgent need to characterize the risk factors and aetiology of acute heart failure in Ghana. The Korle-Bu Teaching Hospital is the premier and largest tertiary hospital in Ghana receiving referrals from all over the country and the sub-region. This study, therefore, presents the cardiovascular risk factors and the underlying aetiology of AHF at the Korle Bu Teaching Hospital.

## Method

### Study design and setting

This was a hospital-based cross-sectional case study recruiting every consecutive patient who was admitted for acute heart failure (AHF) during the study period at the Department of Medicine & Therapeutics (DOMT) of the Korle-Bu Teaching Hospital (KBTH). KBTH is a tertiary referral center located in the national capital of Ghana, Accra with 1600 bed capacity and twelve different departments. The DOMT is a 134-bed facility operating under four teams and has a patient flow of about 300 per month. The department runs a 24-h emergency service and admits patients through the emergency department, outpatient clinics and other specialist clinics. Referrals are received mainly from the southern sector of Ghana but also from all the 16 regional hospitals, district hospitals, private hospitals and specialist clinics. Other referrals are received from polyclinics, maternity homes and some health centers.

### Study population, and inclusion and exclusion criteria

This study involved all adult patients who were admitted and managed for AHF in all four medical wards and the emergency department of the Korle-Bu Teaching Hospital from March to October 2014. Consecutive patients 18 years and above who were admitted with initial diagnosis of AHF and who met the modified Framingham criteria [16] for the diagnosis of HF were included in the study.

#### Major criteria included:

Paroxysmal nocturnal dyspnoea, neck vein distention, pulmonary rales, radiographic cardiomegaly (cardiothoracic ratio > 0.50), acute pulmonary oedema, S3 gallop, and hepatojugular reflux.

#### Minor criteria included:

Bilateral ankle oedema, nocturnal cough, dyspnoea on ordinary exertion, hepatomegaly, pleural effusion, tachycardia (heart rate > 120/min) and weight loss of more than 4.5 kg in 5 days in response to treatment of congestive HF [16]. Minor criteria were acceptable only if they could not be attributed to another medical condition. The diagnosis of HF required that two major or one major and two minor criteria were present concurrently together with echocardiographic evidence of structural heart disease [16].

#### Exclusion criteria

Patients who did not meet the Framingham criteria, were unable to complete full diagnostic evaluation, were pregnant, or were less than 18 years were excluded from the study**.**

### Sample size determination

The sample size for the study was determined using the formula for single cross-sectional survey based on the parameter; earlier studies in Ghana, the expected prevalence (p) of AHF among medical admissions of 8% [11], the standard score (z) of 1.96 at 95% confidence interval, desired margin of error of 5% and a 20% non-response rate. A total of 140 minimum sample size was determined.

### Data collection and case definition

A standard questionnaire which was administered by the principal investigator (a cardiologist) and a trained research assistant (a physician) was used to collect data including demographic characteristics (including age, sex, ethnicity, religion, occupation, educational status and marital status), presenting complaints, and symptoms of HF, history of hypertension, diabetes, dyslipidaemia, smoking history, drug history and previous diagnosis of HF, and family history of heart diseases after informed consent was obtained. Clinical examination was done by the first author (a cardiologist), guided by modified Framingham criteria [16] and the New York Heart Association functional classification [17] and senior cardiologists, for all patients within 24 h after admission, with patients inclined at 45^0^inclinations unless the level of dyspnoea did not permit this position, in which case the examination was done in a sitting position. Physical examination was done with emphasis on looking for signs of HF and pointers to probable causes and risk factors. Physical examination assessed the Heart rate, pulse character, jugular venous pressure, Body mass index (BMI), blood pressure (BP), presence of third heart sound (S3), Hepatomegaly, Hepatojugular reflux, bilateral basal crepitations and pitting ankle oedema.

A standard postero-anterior Chest X-ray was done by a certified radiology technologist, and examined by the first author for cardiomegaly, pulmonary oedema (alveolar and interstitial oedema), pleural effusion, consolidation, fibrosis and other lung diseases. Echocardiographic and electrocardiographic data were collected within five days of admission. All the patients had 2D, M-mode, and Doppler Transthoracic echocardiography (TTE) as part of a comprehensive evaluation done by the first author (a cardiologist) with expert guidance from supervisors (experienced cardiologists) and in accordance with standard guidelines, e.g., [18, 19] using Mindray Diagnostic Ultrasound System model DC-6 equipped with 3.5 MHz cardiac transducer. A resting 12-lead ECG was recorded for all patients at presentation using Schiller AT-10 12-channel ECG machine at a speed of 25 mm/s and a calibration of 10 mm = 1 mV in accordance with the American Heart Association/American College of Cardiology Foundation/Heart Rhythm Society recommendations for the standardization and interpretation of the electrocardiogram [20]. Fasting blood sugar and/or glycated haemoglobin (HBA1c) level was done by venous blood sampling the morning after admission (ensuring at least 12 h of fast over sleep period). All other laboratory investigations were done within the index admission, which included fasting serum lipids, full blood count (FBC), urinalysis, blood urea, creatinine and electrolytes. The clinical presentations of acute health failure are presented elsewhere.

### Data analysis

Data collected were entered, cleaned and analyzed using SPSS version 20 software. Descriptive analysis of the cardiovascular risk factors and underlying aetiology of heart failure was done. Continuous variables were summarized using mean and measure of spread using standard deviation and range were also calculated. Chi-square and Fischer exact tests where appropriate were done to compare the prevalence of cardiovascular risk factors and major underlying aetiologies of HF by gender and age.

## Results

The mean age of the study participants was 51.3 ± 16.8 years. There were more males (56.4%) than females (43.6%). Patients were mostly urban settlers (78.6%). The socio-demographic characteristics of the patients are presented in Table 1.

**Table 1:** Demographic characteristics of study participants

Characteristics	Frequency	Percent
Age (years)		
< 40	35	25.0
≥ 40	105	75.0
Sex		
Male	79	56.4
Female	61	43.6
Ethnicity		
Akan	58	41.4
Ga	34	24.3
Ewe	26	18.6
Others	22	15.8
Residence		
Rural	30	21.4
Urban	110	78.6
Religion		
Christian	125	89.3
Others	15	10.7
Marital status		
Never married	26	18.6
Married	85	60.7
Separated/divorced/widowed	29	20.7
Occupational status		
Unemployed	14	10.0
Employment	126	90.0
Educational status		
No formal education	17	12.1
Primary	50	35.7
Secondary	41	29.3
Tertiary	32	22.9

### Prevalence of cardiovascular risk factors among Heart failure patients

Table 2 presents the cardiovascular risk factors among the patients. The commonest cardiovascular risk factors observed among participants were hypertension (46.5%), history of previous HF (40.7%), excessive alcohol use (38.6%), family history of heart disease (29.3%) which was predominantly hypertension (68.3%), and more than half of the patients presented with multiple risk of 2 or more factors (87.1%).

**Table 2:** Cardiovascular risk factors for acute heart failure among study participants

Risk factors	Freq (%)	Sex, n (%)		*p* value	Age		*p* value
		Male	Female		< 40 yrs	≥ 40 yrs	
Ever smoked	20 (14.3)	19 (95.0)	1 (5.0)	< 0.001*^¥^	3 (15.0)	17 (85.0)	0.191^¥^
Current smoking	7 (5.0)	6 (85.7)	1 (14.3)	0.137^¥^	1 (14.3)	6 (85.7)	0.673^¥^
Alcohol use	54 (38.6)	46 (85.2)	8 (14.8)	< 0.001*	12 (22.2)	42 (77.8)	0.239
Current Alcohol use	28 (20.0)	24 (85.7)	4 (14.3)	< 0.001*^¥^	9 (32.1)	19 (67.9)	0.572
History of diabetes	22 (15.7)	11 (50.0)	11 (50.0)	0.508	1 (4.5)	21 (95.5)	0.008*^¥^
History of hypertension	65 (46.4)	37 (56.9)	28 (43.1)	0.913	9 (13.8)	56 (86.2)	0.001*
History of Dyslipidaemia	20 (14.3)	9 (45.0)	11 (55.0)	0.266	3 (15.0)	17 (85.0)	0.191^¥^
BMI							
< 18.5	11 (7.9)	218.2)	9 (81.8)		6 (54.5)	5 (45.5)	
18.5–24.9	60 (42.9)	43 (71.7)	17 (28.3)	< 0.001*^¥^	19 (31.7)	41 (68.3)	0.063^¥^
25.0–29.9	45 (32.1)	27 (60.0)	18 (40.0)		11 (24.4)	34 (75.6)	
30 and above	24 (17.1)	7 (29.2)	17 (70.8)		3 (12.5)	21 (87.5)	
Previous history of heart failure	57 (40.7)	32 (56.1)	25 (43.9)	0.955	14 (24.6)	43 (75.4)	0.471
Family history of heart disease	41 (29.3)	23 (56.1)	18 (43.9)	0.959	9 (17.1)	32 (82.9)	0.316
Specific family history of heart disease							
Hypertension	28 (68.3)	13 (46.4)	15 (53.6)		6 (21.4)	22 (78.6)	
Diabetes Mellitus	2 (4.9)	2 (100.0)	0 (0.0)		0 (0.0)	2 (100.0)	
Stroke	3 (7.3)	3 (100.0)	0 (0.0)	0.229^¥^	1 (33.3)	2 (66.7)	0.858^¥^
HPT + DM	7 (17.1)	4 (57.1)	3 (42.9)		2 (28.6)	5 (71.4)	
Cardiomyopathy	1 (2.4)	1 (100.0)	0 (0.0)		0 (0.0)	1 (100.0)	
Multiple risk*							
None	18 (12.9)	7 (38.9)	11 (61.1)	0.068	8 (44.4)	10 (55.6)	0.009*
2 or less	50 (35.7)	25 (50.0)	25 (50.0)		19 (38.0)	31 (62.0)	
3 or more	72 (51.4)	47 (65.3)	25 (34.7)		12 (16.7)	60 (83.3)	

*p < 0.05, ^¥^*p *values from Fischer exact test, *multiple risks combines risk from ever and current smoking, previous and current alcohol use, BMI, history of diabetes, hypertension, dyslipidaemia and previous heart failure and family history of heart disease

The history of hypertension (86.2% vs 13.8%; *p* = 0.001), diabetes (95.5% vs 4.5%; *p* = 0.008) and multiple risk of 3 or more factors (83.3% vs 16.7%; *p* = 0.009) were significantly prevalent among patients aged 40 years and above compared to those aged below 40 years. Male patients compared to female patients had high prevalence of smoking (95.0% vs 5.0%; *p* < 0.001), and alcohol use (85.2% vs 14.8%; *p* < 0.001). However, more females than males (70.8% vs 29.2%; *p* = 0.001) had BMI of 30 kg/m^2^ and above.

### Underlying aetiology of heart failure

Table 3 shows the main underlying aetiology of acute heart failure among patients. The major causes of AHF were dilated cardiomyopathy (38.6%), hypertensive heart disease (21.4%), ischaemic heart disease (13.6%) and valvular heart disease (12.9%). The commonest cause of dilated cardiomyopathy identified among patients was idiopathic (68.3%) and patients with valvular heart disease were mostly regurgitant valvular lesions.

**Table 3:** Distribution of common underlying aetiology of AHF by gender and age among study participants

Aetiology	n(%)	Sex, n (%)		*p* value	Age in years, n (%)		*p* value	Multiple risk		
		Male	Female		< 40	≥ 40		< 2	≥ 2	*p* value
Hypertensive heart disease	30 (21.4)	20 (66.7)	10 (33.3)	0.696	6 (20.0)	24 (80.0)	0.004*^¥^	4 (13.3)	26 (86.7)	< 0.001*^¥^
Dilated cardiomyopathy	54 (38.6)	30 (55.6)	24 (44.4)		20(37.0)	34 (63.0)		25 (46.3)	29 (53.7)	
Ischemic heart disease	19 (13.6)	11 (57.9)	8 (42.1)		0 (0.0)	19 (100)		1 (5.3)	18 (94.7)	
Valvular heart disease	18 (12.9)	9 (50.0)	9 (50.0)		8 (44.4)	10 (55.6)		8 (47.4)	10 (55.6)	
Other^ф^	19 (13.6)	9 (47.4)	10 (52.6)		5 (26.3)	14 (73.7)		9 (47.4)	10 (52.6)	

^ф^other include Cor pulmonale, Congenital heart disease, Thyrotoxic heart disease, Pericardial disease, Endomyocardial fibrosis, Hypertrophic cardiomyopathy, Atrial myxoma

*p ≤ 0.05, ^¥^*p *values from Fischer exact test, multiple risks combines risk from ever and current smoking, previous and current alcohol use, BMI, histories of diabetes, hypertension, dyslipidaemia and previous heart failure and family history of heart disease

### Comparison of the major causes of HF by gender, age, and multiple risk factors

Table 3 presents the comparison of underlying aetiology of acute heart failure by gender and age and multiple risk factors. There were no significant differences in the underlying aetiology of HF between male and female patients. However, patients aged 40 years and above (*p* = 0.004) and presenting with multiple risk of 2 or more factors (*p* < 0.001) were diagnosed more with hypertensive heart disease, dilated cardiomyopathy ischaemic heart disease and valvular heart disease compared to those aged less than 40 years and presented with one or no risk factor respectively.

## Discussion

The aim of the study was to examine the prevalence of cardiovascular risk factors and underlying aetiology of acute heart failure at the Korle-Bu Teaching Hospital, the national referral center serving the southern sector of Ghana.

The study found that hypertension was the most common risk factor for AHF. This is consistent with several studies done in Ghana [13, 14, 21] and other African countries [22–24]. Hypertension was most prevalent in patients aged 40 years and above. This finding is similar to the finding of a previous study where systolic blood pressure markedly increased with age [25]. A meta-analysis of several studies in Ghana has shown that hypertension detection, treatment and control rates are very poor [26]. This is due to several factors including poverty, illiteracy, poor accessibility to healthcare services and traditional beliefs about medication. In addition, medication non-adherence rates among patients with hypertension have been shown to be very high in a recent study [27]. This begs for urgent action to improve hypertension detection, treatment and control rates in Ghana if the raging epidemic of HF is to be curtailed. The study found other risk factors including history of smoking, alcohol use, diabetes mellitus, previous heart failure, dyslipidaemia, and obesity, similar to the findings of previous studies [25, 28]. Smoking and alcohol use were significantly higher in males whereas obesity (BMI of > 30 kg/m^2^) was significantly higher in females and those aged 40 years and above. This is consistent with the findings made in a previous study where the history of former or current smoking was frequent among male patients and female patients were more obese [28]. The current study found that the prevalence of diabetes mellitus was significantly higher in patients aged 40 years and above. This is in line with what has been reported elsewhere where the prevalence of DM was high among middle-age patients presenting with acute heart failure [25]. Cardiovascular risk factors particularly dyslipidaemia, hypertension, diabetes mellitus and smoking are widespread in adults worldwide [29], which may possibly be due to urbanization, increase life expectancy and changes in lifestyles including unhealthy diets and lack of physical activity.

The study identified cardiomyopathies and hypertensive heart disease as the most common underlying aetiology of HF in this study. Other common underlying aetiology were ischaemic and valvular heart diseases. This is similar to the findings of the studies by Owusu et al. in Komfo Anokye Teaching Hospital in Kumasi [14, 15], and Amoah et al. [13] in the National Cardiothoracic Center in Accra. However, in this study fewer rheumatic heart diseases and more cardiomyopathies and ischaemic heart diseases were identified compared to the earlier studies. This probably reflects the changing lifestyle of Ghanaians and the growing urbanization, ageing population and adoption of a westernised diet and lifestyle. Dilated cardiomyopathy, the most frequent aetiology in this study, has been shown to be a common cause of HF in sub-Saharan Africa from earlier studies [30, 31]. Several important causes of dilated cardiomyopathy have been identified in sub-Saharan Africa including HIV cardiomyopathy, peripartum cardiomyopathy, myocarditis, alcohol-induced cardiomyopathy and genetic/familial forms [31]. Familial disease accounts for 30–50% of cases depending on the extent to which investigations are carried out to identify the underlying cause. The current study identified idiopathic dilated cardiomyopathy (65.1%) as the commonest form of dilated cardiomyopathy comparable to the 50% identified by Felker et al. [32] among 1230 patients evaluated with initially unexplained cardiomyopathy. The high proportion of idiopathic dilated cardiomyopathy observed in the current study could probably be because of incomplete investigations in this study. Echocardiography is not very sensitive in differentiating the underlying causes of dilated cardiomyopathy. Furthermore, cardiac MRI and coronary angiography were not performed so one is likely to misclassify some cases of ischaemic heart disease as dilated cardiomyopathy and vise versa. Similarly, viral screening and serological tests were not done in this study. In the study by Felker et al. [32], the prognosis of patients with ischaemic cardiomyopathy, infiltrative cardiomyopathy and HIV-associated cardiomyopathy was poorer than those with idiopathic dilated cardiomyopathy. It is therefore of utmost importance to fully evaluate HF patients with dilated cardiomyopathy for any probable underlying causes.

Hypertensive heart disease results from chronic systemic arterial hypertension and has emerged as the commonest cause of AHF in several studies in Africa [6, 22, 28]. The causes of hypertensive heart disease seem to be a likely gene-environment interaction whereby weight gain, high salt intake and psychosocial factors may facilitate the rapid development of hypertension and hypertensive heart disease in susceptible individuals [33]. It is clear from this study that hypertension remains a major health challenge in Ghana and other studies have shown that it still remains undiagnosed and poorly controlled in a majority of the population [26].

Furthermore, ischaemic heart disease was found as more common than primary valvular heart disease in the aetiology of HF. This finding is at variance with the findings in earlier studies in Ghana [13, 15], the heart of Soweto [7] and the THESUS-HF studies [6]. In the national capital of Ghana, several studies have shown a high prevalence of traditional cardiovascular risk factors for coronary artery disease; such as hypertension, obesity, diabetes mellitus, dyslipidaemia and sedentary lifestyle [17, 26, 34]. This could therefore account for ischaemic heart disease becoming common as observed in this current study.

Our current study found only 12.9% of the AHF was due to primary valvular heart disease. Moreover, most of the valvular heart diseases were degenerative rather than rheumatic, reflecting the ageing population. A similar low prevalence of rheumatic heart disease as a cause of contemporary HF was found in Abeokuta, Nigeria in a recent registry in which only 2.4% of the 452 HF patients had rheumatic heart disease [23]. Both studies were done in urban communities in Africa where living standards and environmental conditions are better, thus may not be a true reflection of the general West African population.

Congenital heart diseases were very few in this study, like that found in the Abeokuta HF registry where only 2 congenital heart disease cases were identified in the 452 adults with HF [23]. This may either be due to patients sticking to their pediatric doctors because of long-term relationships or some of them not living long enough into adulthood as a result of the lack of resources for corrective heart surgeries in most hospitals in sub-Saharan Africa. However, congenital heart diseases have been identified as very common causes of heart failure in earlier studies [13, 14]. This is probably due to the differences in the age range of patients included in the studies. Whereas Amoah et al. [13] included patients 4 months to 95 years, in our current study, only adults 18 years and above were recruited.

The study found that hypertensive heart disease, cardiomyopathies, ischaemic and valvular heart diseases were predominant in patients aged 40 years and above and those presenting with multiple risk factors. This is consistent with the finding made in a similar study among heart failure patients in a tertiary hospital in Kumasi, Ghana [14]. It has been stipulated that enhancements in public health have led to the high prevalence of chronic diseases among the ageing populations [35].

## Study limitation

A major limitation of this study is the inability to do natriuretic peptides on admission. This test was not routinely available in the facility during the time of the study. Diagnosis of ischaemic cardiomyopathy was based on history, electrocardiography, and echocardiography with no coronary angiography. Additionally, virological and serological tests were not done to investigate the underlying cause of dilated cardiomyopathies. A previous history of heart failure was established mainly by clinical history and may suffer from recall bias. The definition of hypertensive heart disease used for this study might have missed out patients with end-stage hypertensive heart disease with thinned walls.

## Conclusion

The study has shown clearly that the major underlying aetiologies of acute heart failure in adults admitted to Korle-Bu Teaching Hospital were dilated cardiomyopathy, hypertensive heart disease, ischaemic heart disease and valvular heart disease irrespective of gender. Dilated cardiomyopathy and hypertensive heart disease were the most common aetiologic factors, especially in patients aged 40 years and above and those presenting with multiple risk factors. Whilst efforts at controlling rheumatic heart disease and other infections should continue, equally greater policy attention should be given to non-communicable diseases like hypertension, diabetes, and coronary artery disease to reduce heart failure burden in Ghana.

## Data Availability

The dataset generated during the current study is not publicly available due to issues of confidentiality but are available from the corresponding author on reasonable request.

## Abbreviations

HF: Heart failure
KBTH: Korle Bu Teaching Hospital
BMI: Body mass index
HIV: Human immunodeficiency virus
DOMT: Department of Medicine & Therapeutics
